# Pathology and Treatment of Scarlet Fever

**Published:** 1870-10

**Authors:** 


### Miscellaneous.

### Pathology and Treatment of Scarlet Fever.

Dr. Renfrew, of Glasgow, read a paper on this subject before the British Medical Association. He stated that scarlet fever is one of the zymotic diseases, which diseases are produced by an organized substance entering the body, which has the power of multiplying itself. In multiplying itself the blood is disordered, the nervous system deranged, the circulation quickened, and the secretions and excretions are changed. The poisons of the zymotic disease are not thrown off by the usual eliminating organs, but each poison is eliminated by a particular part of the body—smallpox by the skin, cowpox at the point of introduction, enteric fever by the lower part of the ileum, scarlet fever by the fauces and nose. When the poisons are thrown off there is always irritation and inflammation. As the poison of scarlet fever is thrown off by the fauces and nose, a large portion must pass into the stomach to be reabsorbed, intensifying and prolonging the disease. The remedies given in scarlet fever should be those that will destroy the poison; moderate and assist physiological changes. To accomplish these ends a mixture of chlorate of potash and tincture of steel is given, which contains chlorine, muriatic acid, iron, and chlorate of potash. The chlorine destroys the poision; the acid supplies acid to the blood, which is in a subacid condition; the iron improves the red disks, which are in a black and melanosed condition; the chlorate of potash supplies oxygen, to assist in oxidizing the disintegrated material that is floating in the blood.—*Lancet,* August 20, 1870.

